# Bridging the gap: the need for community-engaged research on blood cancer self-management experiences among South Asians

**DOI:** 10.1007/s00520-025-10154-z

**Published:** 2025-11-22

**Authors:** Sandeep Dhillon, Ailsa C. Sirois, Deepjot Sanghera, Anmol Kaur, Aperrna Sasheendren, Hira Mian, Lorelei Newton, Shahid Ahmed, Theodore D. Cosco, Deepesh Lad, Cara Bradley, Martine Puts, Kristen R. Haase

**Affiliations:** 1 School of Nursing, University of British Columbia, Vancouver, British Columbia Canada; 2 Vancouver Community College, Vancouver, British Columbia Canada; 3 Faculty of Health Sciences, McMaster University, Hamilton, Ontario Canada; 4 Department of Oncology, University of Saskatchewan, Saskatoon, Saskatchewan Canada; 5 Saskatchewan Cancer Agency, Saskatoon Cancer Center, Saskatoon, Saskatchewan Canada; 6 School of Public Policy, Simon Fraser University, Vancouver, British Columbia Canada; 7 Oxford Institute of Population Ageing, University of Oxford, Oxford, UK; 8 Division of Hematology, Department of Medicine, Leukemia/BMT Program of BC, Vancouver, British Columbia Canada; 9 University of British Columbia, Vancouver, British Columbia Canada; 10 Archer Library, University of Regina, Regina, Saskatchewan Canada; 11 Lawrence Bloomberg Faculty of Nursing, University of Toronto, Toronto, Ontario Canada; 12 Department of Supportive Care, Princess Margaret Cancer Centre, Toronto, Ontario Canada; 13 BC Cancer, Vancouver, BC Canada

Blood cancers, including leukemia, lymphoma, and myeloma, represent a significant burden on global health [1, 2]. In 2020, hematologic malignancies represented approximately 6.2% of all new cancer diagnoses globally, with an estimated 474,519 cases of leukemia (2.5%), 544,352 cases of non-Hodgkin lymphoma (2.8%), and 176,404 cases of multiple myeloma (0.9%) [2]. While substantial advancements have been made in understanding and treating these diseases, disparities in health service use and self-management experiences between ethnic groups remain a prominent concern, particularly among South Asians. “South Asian” refers to individuals of Bangladeshi, Indian, Indo-Caribbean, Pakistani, Sri Lankan, Nepali, Bhutanese, and Maldivian descent [3, 4]. Although unified by geographic origin, South Asians represent a diverse population with variations in the languages they speak, the religions they practice, and the sociocultural norms they hold [5]. Given their unique sociocultural context, they also experience barriers to healthcare access, such as language barriers, stigma, cultural and religious beliefs, and gender roles. Therefore, investigating self-management practices among this population is essential for promoting equitable cancer care [6].

Self-management is recognized as a critical component of managing chronic conditions, navigating symptoms, treatment side effects, and psychosocial challenges [7–9]. In the context of cancer, self-management tasks typically include medication adherence, symptom monitoring, lifestyle adjustments, and healthcare navigation. However, for South Asians, self-management may extend beyond individual agency, shaped by familial, community, and spiritual influences. Therefore, a more culturally bound definition of self-management is: “the ability of the individual, in conjunction with family, community, and healthcare professionals, to manage symptoms, treatments, lifestyle changes, and psychosocial, cultural, and spiritual consequences of health conditions (p. 1145) [10].”

While self-management has been explored in South Asians with diabetes, cardiovascular disease, and hypertension [11–13], these studies indicate that such practices are significantly shaped by cultural, familial, and religious norms. For instance, Navodia et al. [11] and Patel et al. [13] found that South Asian patients with diabetes often depended on their social networks, specifically their family members, for emotional support, meal planning, and taking their medication consistently. Siddique et al. [12] noted that fatalistic and spiritual beliefs commonly shaped South Asian patients’ perceptions of disease control and adherence to cardiovascular treatment. These findings highlight barriers such as health literacy gaps, reliance on familial decision-making, and cultural perceptions of illness. They also suggest that self-management among South Asians is a socially embedded process rather than solely an individual responsibility. However, little is known about how these factors influence blood cancer self-management. Understanding these practices is critical to informing culturally responsive supportive care interventions for South Asians.

To address this gap in the literature, we conducted a scoping review, where the primary objective was (1) to map the current evidence on self-management practices among South Asians with blood cancer; (2) to identify challenges, barriers, and facilitators to self-management within this population; (3) to highlight gaps in the literature that warrant further exploration to support culturally tailored care.

We followed the framework proposed by Arksey and O’Malley [14], incorporating refinements by Levac et al. [15] and adhering to PRISMA scoping review guidelines [16]. We then conducted a comprehensive search across PubMed, CINAHL, and Embase databases from inception to October 16, 2024, in consultation with an academic librarian (Cara Bradley). The search strategy included studies conducted in English, Punjabi, and Hindi and a combination of terms such as “self-management,” “South Asian,” “blood cancer,” and “social support.” The titles and abstracts, and full-text screening were conducted independently by two reviewers using Covidence [17]. Discrepancies were resolved through discussion. Although our search included literature related to self-management practices for South Asians (18+) with blood cancer in Punjabi and Hindi, no studies in these languages were identified.

Of the 7746 studies screened, we identified 63 full-text articles for review. However, none fully met our inclusion criteria, which required studies to focus on South Asian adults (18+) diagnosed with any type of blood cancer, with at least 50% of participants having blood cancer or a subanalysis available, and to examine self-management experiences, strategies, or interventions. The search and screening process is presented in the PRISMA-ScR flow diagram (Fig. 1). The diagram outlines the number of studies identified, screened, excluded, and the reasons for exclusion. While some studies briefly mentioned aspects of self-management in their discussion sections, no studies had results related to self-management or provided an examination of self-management experiences and practices, culturally tailored interventions, or community-informed strategies. This lack of research strongly indicates that South Asians with blood cancer may be navigating self-management challenges with limited to no culturally adapted guidance or support. Without empirical evidence, health and social care systems may be neglecting the needs of this group. Such neglect reinforces disparities in supportive cancer care.

**Fig. 1:**
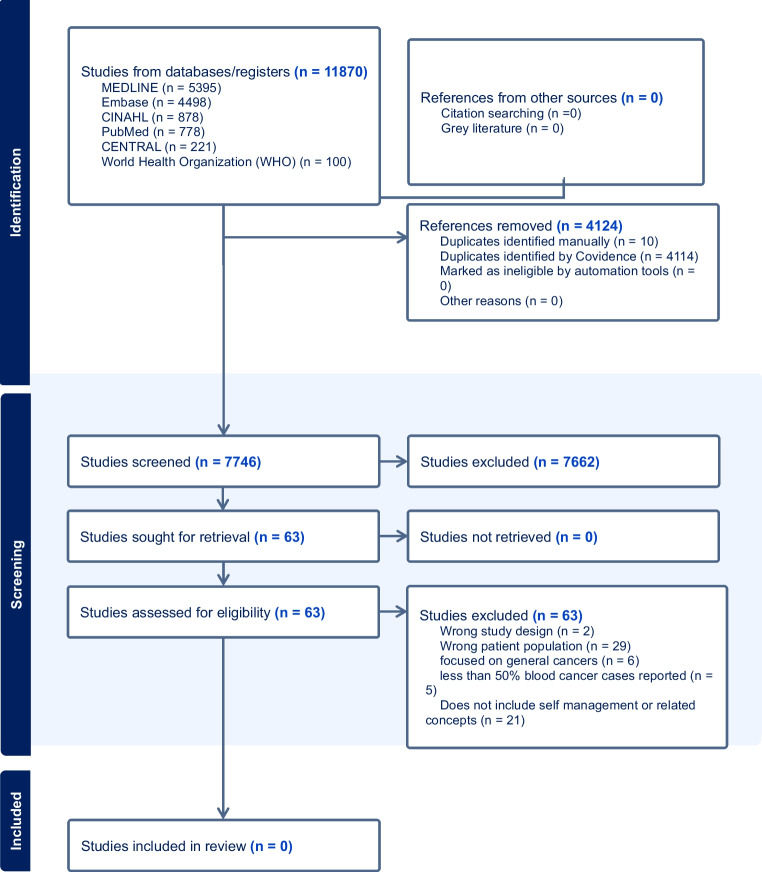
PRISMA diagram

In this commentary, we highlight the implications of this gap, discuss the critical role of community-engaged research in addressing the needs of blood cancer self-management among South Asians, and outline future research directions. Our work emphasizes the importance of collaboration with South Asian communities to explore and develop culturally responsive self-management strategies.

More specifically, there is a critical gap in studies investigating blood cancer self-management experiences among South Asians, with existing research yet to explore their experiences. Without this foundational knowledge, it is unclear whether current self-management interventions are effective or culturally appropriate for this population. Given this lack of evidence, a traditional top-down research approach, where interventions are developed based on existing literature, is not feasible. Rather, engaging with South Asian communities is necessary to co-produce insights and design culturally informed self-management strategies [18].

Community-engaged research involves building partnerships with trusted community and religious organizations to identify community leaders and gatekeepers. By leveraging local knowledge, community members and researchers can collaboratively co-develop the study design and determine appropriate data collection methods (i.e., interviews, storytelling, focus groups, or other culturally meaningful approaches) [18]. This collaborative process ensures cultural relevance, centers lived experiences, and fosters mutual learning and trust. As a result, community-engaged research will help: (1) amplify the voices of South Asians with blood cancer, ensuring that the research reflects their lived experiences; (2) address cultural barriers and facilitators, such as stigma, religious beliefs, and family dynamics, which may influence self-management experiences; (3) enhance community participation and foster trust and acceptability of culturally relevant cancer care programs. Without community engagement, supportive care frameworks may remain Eurocentric and inaccessible. These models often prioritize Western individualistic norms of autonomy, such as goal-setting and self-directed care plans [19]. However, given the collectivistic and family-centered values among South Asians, these frameworks may overlook their cultural needs and contribute to suboptimal cancer outcomes.

To address this urgent gap, we aim to partner with South Asian community members, organizations, and stakeholders to explore the lived experiences of blood cancer self-management, identify key barriers and facilitators, and co-develop strategies that align with sociocultural norms and beliefs. Using a community-engaged approach, we will conduct qualitative interviews to address this research void and inform future intervention studies. By including South Asian community members and gatekeepers, we ensure that findings translate into meaningful, real-world improvements in culturally competent cancer care.

The lack of research on blood cancer self-management in South Asians highlights critical gaps in equitable cancer care. Structural barriers, limited culturally tailored interventions, and systemic disparities in supportive oncology persist [20, 21]. These ongoing inequities highlight the need for targeted solutions. To address this gap, our next phase will focus on community-driven research to co-design culturally competent self-management strategies that align with global efforts to reduce cancer care disparities [9]. We will prioritize understanding how sociocultural values and beliefs, such as family roles, religious beliefs, language, and stigma, influence self-management. Potential challenges include building trust with South Asian communities, who may be unfamiliar with research, recruiting diverse participants across South Asian subgroups, and ensuring cultural relevance across contexts. By integrating patient, caregiver, and provider perspectives, we aim to develop inclusive, sustainable interventions that promote equity, diversity, and accessibility in supportive oncology care.

## Data Availability

No datasets were generated or analysed during the current study.
